# Adjuvanticity of Tannic Acid-Modified Nanoparticles Improves Effectiveness of the Antiviral Response

**DOI:** 10.2147/IJN.S512509

**Published:** 2025-04-01

**Authors:** Martyna Janicka, Marcin Chodkowski, Aleksandra Osinska, Klaudia Bylinska, Oliwia Obuch-Woszczatyńska, Magdalena Patrycy, Grzegorz Chodaczek, Katarzyna Ranoszek-Soliwoda, Emilia Tomaszewska, Grzegorz Celichowski, Jaroslaw Grobelny, Joanna Cymerys, Małgorzata Krzyżowska

**Affiliations:** 1 Division of Medical and Environmental Microbiology, Military Institute of Hygiene and Epidemiology, Warsaw, Poland; 2 Division of Microbiology, Department of Preclinical Sciences, Institute of Veterinary Medicine, Warsaw University of Life Sciences, Warsaw, Poland; 3 Laboratory of Parasitology, Military Institute of Hygiene and Epidemiology, Warsaw, Poland; 4 Division of Pharmacology and Toxicology, Department of Preclinical Sciences, Institute of Veterinary Medicine, Warsaw University of Life Sciences, Warsaw, Poland; 5 Łukasiewicz Research Network – PORT Polish Center for Technology Development, Life Sciences and Biotechnology Center, Wroclaw, Poland; 6 University of Lodz, Faculty of Chemistry, Department of Materials Technology and Chemistry, Lodz, Poland

**Keywords:** HSV-1, AgNPs, AuNPs, tannic acid, microglia

## Abstract

**Introduction:**

Herpes simplex virus type 1 (HSV-1) causes recurrent infections of skin and mucosal tissues with high global prevalence. HSV-1 also invades the nervous system where it establishes a lifelong latency-making infection poorly treatable We previously showed that both tannic acid-modified silver and gold nanoparticles (TA-Ag/AuNPs) inhibit HSV-1 infection in vitro.

**Methods:**

We used an in vitro and in vivo model of HSV-1 infection to study how metal type, size and tannic acid modification of nanoparticles can influence development of the early innate response and the mounting of specific anti-HSV-1 response upon treatment of the nasal mucosa.

**Results:**

We found that tannic acid is necessary for binding with HSV-1, with smaller sizes independent of the NPs composition, whereas for larger NPs, only TA-AgNPs can inhibit HSV-1 infection. Intranasal treatment of HSV-1 infection with TA-Ag/AuNPs results in lower viral titers and a better antiviral response, followed by increased IFN-α, CXCL9, and CXCL10 levels as well as infiltration of T cells and NK cells in the infected sites. We also found that the application of TA-NPs to the nasal cavities of infected mice induced infiltration of both monocytes and Langerhans cells (LCs), which lasted longer compared to the application of unmodified NPs. Furthermore, TA-NPs activated monocytes and microglia to produce antiviral cytokines and chemokines better than unmodified NPs, except for the large TA-AuNPs.

**Discussion:**

Treatment of the mucosal tissues at the early stage of HSV-1 infection helps to modulate specific and effective antiviral immune response by attracting cytotoxic lymphocytes and inducing the production of antiviral cytokines and chemokines. Furthermore, tannic acid modification is helpful for the removal of nanoparticles from the respiratory tract, which increases the safety of nanoparticle applications to treat infections.

## Introduction

Recently, nanotechnology has been gaining much attention as a useful tool for development of alternative methods for infection treatment.^1^,^2^ The highly efficient lipid nanoparticle (LNP)‐based RNA vaccines for COVID‐19 are the best example of nanotechnology use in infection prophylaxis. General antiviral mechanisms of nanoscale interventions should include: (a) inactivation of the virus by a direct binding, formation of complex bonds and electrostatic interaction (b) blockage of the host receptors, such as heparan sulfate (HS) and sialic acid (SA); and (c) intracellular delivery of antivirals against viruses.^1–5^ Several nanomaterials, including Au and Ag-based metal nanoparticles, have demonstrated virucidal effects based on their specific physiochemical properties. They can directly bind with the viral envelope or capsid proteins, resulting in the loss of the virus infectivity, block its binding to receptors, or exert a toxic effect inside the infected cells to inhibit viral replication.^1–5^

Furthermore, nanoparticles can stimulate the innate immune response by mimicking the naturally existing pathogen-associated molecular patterns (PAMPs) recognized by Toll-like receptors (TLRs) present on variety of immune cells, including antigen presenting cells (APC), as well as other innate immune cells, such as neutrophils, mast cells, and NK cells.^6^ Additionally, nanoparticles can protect, stabilize, and present foreign particles (antigens) to APCs such as macrophages, dendritic cells (DCs), and B cells.^7–9^ Macrophages and DCs exhibit strong ability to phagocyte antigens; they can capture nanoparticles loaded with antigens present in the blood circulation and tissues, and accumulate them intracellularly.^9–11^ Metal nanoparticles have been shown to act as adjuvants by modulating the immunogenicity of the antigen through various mechanisms.^8^,^10^,^11^ Despite the increasing use of over-the-counter products containing solid metal-based nanoparticles to treat skin and mucosal infections, there is very little concern about the correlation between modification, size, and ability of NPs to stimulate local immunity.

Human herpesvirus type 1 (HSV-1) infects humans mainly via oral epithelium, conjunctiva, or genital mucosa.^12^,^13^ The virus establishes latency in the trigeminal ganglia resulting in a life-long infection. HSV-1 infection can result in herpes simplex encephalitis (HSE) and is related with neurodegenerative processes.^12^,^13^ Due to its ability to induce latency in neuronal ganglia, the virus is poorly visible to immune competent cells, and it is difficult to develop an effective vaccine.^12^,^13^ We have previously demonstrated that treatment of early mucosal HSV type 1 or 2 infection with 30 nm epigallocatechin gallate (EGCG)-modified AgNPs induces infiltration of dendritic cells and monocytes to the infected sites, followed by infiltration of CD8+ T cells and NK cells altogether resulting in lower viral loads in tissues of the treated mice.^14^ We also found that tannic acid-modified AgNPs (TA-AgNPs) sized 13, 33 and 46 nm were able to reduce HSV-2 infection in both in vitro and in vivo models.^15–18^ Furthermore, upon mucosal re-challenge with HSV-2, mice treated previously with 30 nm TA-AgNPs showed better induction of effector-memory CD8+ T cells and higher titers of anti-HSV-2 neutralization antibodies.^16^

In this work, we compared tannic acid-modified AgNPs and AuNPs of two sizes, 5 and 30 nm for their antiviral properties, using in vitro models of neuronal tissues. We also studied how tannic acid modification, metal type, and size can influence the activation of the early innate response and mounting of the anti-HSV-1 response upon treatment of the nasal mucosa early during infection.

## Material and Methods

### Nanoparticles

To synthesize aqueous colloids of gold and silver nanoparticles with the size of metallic core of 5 nm and 30 nm, respectively, we used a chemical reduction method. The synthesis of metallic nanoparticles was performed using the following chemicals from Sigma-Aldrich (St. Louis, MO, USA): gold (III) chloride hydrate, tannic acid (C_76_H_52_O_46_), sodium citrate (C_6_H_5_Na_3_O_7_ × 2H_2_O), silver nitrate (AgNO_3_), sodium borohydride (NaBH_4_).

### Citrate Metallic Nanoparticles

#### 5 nm AuNPs

A chloroauric acid–water solution (28.986 g, 1.786×10^−2^ wt. %) was poured into a flat bottom flask, then mixed at RT (room temperature). Following addition of sodium borohydride (1.014 mL, 0.5 wt. %), and the solution changed its color to red, indicating formation of AuNPs. The colloid was further mixed for an additional 1 h. After the synthesis process was finished, we adjusted the concentration of sodium citrate to the level present in 5 nm TA-AuNPs colloid by incorporating of 0.296 g of a 4% sodium citrate aqueous solution into the colloid. We were unable to synthesize stable 30 nm AuNPs colloid without the addition of tannic acid as a stabilizer.

#### 5 nm AgNPs

The aqueous solution of sodium citrate (98 g, 2.44×10^−2^ wt. %) was mixed with 1.574 g of AgNO_3_ solution (1%) in the dark on a magnetic stirrer. After 2 min of stirring 0.420 mL of aqueous NaBH_4_ solution (1%) was added dropwise (addition rate 1 drops^−1^). The mixture was further stirred in the dark for 2 h before use.

#### 30 nm AgNPs

We used a two-step procedure to prepare AgNPs with a metallic core size of 30 nm. First, silver seeds were synthesized as follows. The second step of the synthesis procedure included the use of previously obtained silver seeds. The details of synthesis method were given previously.^19^ Since the final concentration of AgNPs was 355 ppm, the colloid was diluted to 100 ppm before use in subsequent experiments.

### TA-Modified Metallic Nanoparticles

The chemical reduction method was also used to prepare the aqueous colloids of gold and silver nanoparticles with the size of metallic core equal to 5 nm and 30 nm and functionalized with tannic. The details of synthesis procedures for TA-AuNPs with the size of 5 nm and 30 nm and TA-AgNPs with the size of 5 nm and 30 nm were previously described.^19^ All colloids were adjusted to the final concentration of 100 ppm for both AuNPs and AgNPs, before the use in subsequent experiments. The tannic acid concentration in the colloids was 0.0315 wt. % (315 ppm).

### Nanoparticles Characterization

The aqueous colloids of AuNPs and AgNPs were characterized by: (a) Dynamic Light Scattering (DLS, Litesizer 500, Particle Analyzer, Anton Paar) and (b) High-Resolution Scanning Electron Microscopy equipped with transmission detector STEM II (HR-STEM, NovaNanoSEM 450, FEI, USA), as previously described.^19^

### Virus and Cells

HSV-1 (strain ID 2762) originated from a biopsy taken from a patient with HSE^20^ (kindly provided by Professor Thomas Bergström of the Department of Virology, University of Gothenburg). The virus was propagated in Vero cells (ATCC^®^ CCL-81), titrated and stored as described previously.^14^ Virus was diluted in ice-cold phosphate-buffered saline (PBS), or in 0.9% NaCl and used within maximum one hour. Vero cells, Neuro-2a cells (ATCC^®^ CCL-131), and RAW-Dual™ cells (InvivoGen, Toulouse, France) were cultured in Dulbecco’s modified Eagle’s medium with GlutaMAX (DMEM), with addition of 10% fetal bovine serum (FBS), 1% penicillin/streptomycin (Thermo Fisher Scientific). RAW-Dual™ cells were cultured with Zeocin^®^ (200 μg/mL) to maintain the secreted embryonic alkaline phosphatase (SEAP) activity and to report NF-κB activation. SEAP activity from the supernatants was measured using QUANTI-Blue™ Solution (InvivoGen, France).

### Primary Cultures

The whole brains of neo-natal C57BL/6 mice were used to establish primary cultures of mixed glial cells or neuronal cells, as described.^14^ Cells obtained by digestion with trypsin (0.025%) were suspended at 10^6^/mL either in mixed glial medium [Dulbecco’s modified Eagle’s/F12 medium with GlutaMAX (DMEM/F12), 10% FBS, 1% penicillin/streptomycin, 5 ng/mL of murine recombinant granulocyte and macrophage colony stimulating factor (GM-CSF), Thermo Fisher Scientific], or in neuronal culture medium (Neurobasal™ with 2% B27 supplement, 1% Glutamax, and 1% penicillin/streptomycin). The primary cultures were maintained 7–10 days before use.

### Antiviral Tests

#### Plaque Reduction

Vero cells cultivated in 24-well plates were used for all tests. TA-modified 5 nm AuNPs, 30 nm AuNPs, 5 nm AgNPs and 30 nm AgNPs as well as unmodified 5 nm AuNPs, 5 nm AgNPs and 30 nm AgNPs at 7.5 μg/mL were mixed with 100 PFU of HSV-1 in 1 mL and incubated for 60 minutes. Subsequently, the mixture was applied to Vero cells as described by Orlowski et al.^15^ After 48 h, cells were stained with 1% crystal violet to visualize plaques before counting their number in each well. The data were presented as the number of plaques per mL (PFU/mL).

#### Post-Entry Treatment

To study how NPs influence the post-entry effect HSV-1 infection, Neuro 2a, primary mixed glial cells and primary neurons were infected at 37 °C. At 6 h post infection (p.i)., infected cells were washed to remove remaining virions before the addition of TA-AgNPs/AuNPs. Analysis of the infected cultures was conducted using qPCR at 24 h p.i.

### CryoTEM

Cryogenic Transmission Electron Microscopy (cryo-TEM) imaging was conducted using a Tecnai F20 X TWIN microscope (FEI Company, Hillsboro, Oregon, USA) equipped with a field-emission gun operating at an acceleration voltage of 200 kV. Images were captured with a Gatan Rio 16 CMOS 4k camera (Gatan Inc., Pleasanton, CA, USA) and processed using Gatan Microscopy Suite (GMS) software (Gatan Inc., Pleasanton, CA, USA). The details were described previously.^14^

Samples were prepared by incubating HSV-1 virus with nanoparticles for approximately 30s, before 3 μL droplet of the suspension was placed a onto the grid, and subjected to further processing and visualization.^14^

### ICP-MS

The samples of tissues collected from the mice were homogenized in PBS (w/v ratio of 1:10). Next, 1 mol/L NaOH solution at a v/v ratio of 1:1 was added and incubated for 3 h at 37 °C. After incubation, samples were centrifuged at 10.000 x g, diluted 500 times and analyzed by ICP-MS, as described^18^ using an Agilent 7900 hPLC-ICP-MS instrument (Agilent Technologies, Santa Clara, CA, USA).

### Quantitative PCR

RNA/DNA Extraction kit (Eurx, Gdansk, Poland) was used to extract DNA/RNA from trigeminal ganglia, noses, and brains. Universal RNA/DNA extraction kit (Eurx) served to isolate DNA/RNA from infected cell cultures. Quantification of HSV-1 was performed by qPCR using primers and a probe for the viral envelope glycoprotein (gB), as described^14–18^ in the QuantStudio™ 5 Real-Time PCR System (Thermo Fisher Scientific) with GoTaq^®^ Probe qPCR Master Mix (Promega, Madison, WI, USA). Data are expressed as HSV-1 copy number per nanogram of total isolated DNA.

RNAs isolated from tissues and cell cultures was converted into cDNA using GoScript™ Reverse Transcriptase (Promega). qPCR reactions for cytokines and chemokines were carried out using GoTaq^®^ Probe qPCR Master Mix (Promega) and TaqMan^®^ probes (Thermo Fisher), as described in^14^ The results were analyzed and presented using the 2−ΔΔCT cycle threshold method.

### Confocal Microscopy

Images of cells and nanoparticles were acquired with an upright Leica SP8 resonant scanning confocal system (Leica Microsystems, Wetzlar, Germany). Mixed glia were fixed with acetone-methanol, dried, then incubated overnight at 4°C with primary antibodies diluted in 2% BSA, 0.1% saponin in PBS. HSV antigens were detected with rabbit polyclonal anti-HSV-1/2 (Dako, Agilent, Santa Clara, CA, USA), microglia were stained with polyclonal goat anti-IBA1 (ThermoFisher Scientific), while to detect astrocytes, we used anti-GFAP (clone 2A5, Abcam). The following secondary antibodies were used: Alexa Fluor^®^ 488^®^ anti-rabbit (HSV), Alexa Fluor^®^ 555 anti-mouse (GFAP), and Alexa Fluor^®^ 647 anti-goat (IBA-1) polyclonal antibodies (ThermoFisher Scientific). The slides were sealed in SlowFade™ Diamond Antifade Mountant containing 4-6-diamidino-2-phenylindole (DAPI; ThermoFisher Scientific). The 63× oil immersion objective (NA 1.40) was used to acquire stacks of confocal 8-bit images with a pixel size of 0.118 µm and a 0.3 µm Z step. The pinhole was set to 1 AU. Nanoparticles were visualized in a laser reflection mode using a 552 nm laser line. Acquisition was performed in the sequential mode to avoid spectral overlap. Images were processed using the Fiji/ImageJ software (National Institutes of Health, USA). First, median filtering (radius of two) was applied to all optical sections from a given Z-stack to reduce noise. Next, maximum-intensity projections encompassing 2–10 optical sections of all channels were created to extract the nanoparticle signal from the imaged volume in a 2D image. The overlap of nanoparticle signal with GFAP- or Iba-1-positive cells was calculated using Mander’s Colocalization Coefficients in the Imaris x64 9.5.1 software (Oxford Instruments, Abingdon, UK) upon smoothing GFAP and Iba-1 channels (Gaussian Blur with a radius of 5) and thresholding (Huang algorithm for GFAP/Iba-1 and MaxEntropy algorithm for the nanoparticle channel).

### In vivo Infection Model

HSV-1 infection model was applied to 8-week-old C57BL/6 mice, as described in.^14^ The protocol for the animal study was approved by the Local Committee on the Ethics of Animal Experiments in Warsaw, Poland (permit Number: WAW2/69/2021). The study was performed in strict accordance with the recommendations of the Polish Act of 21 January 2005 on animal experiments (OJ no 33, item 289) and Directive 2010/63/EU of the European Parliament and the Council of 22 September 2010 on the protection of animals used for scientific purposes. Shortly, mice were anesthetized as described in,^14^ and a total dose of 1×10^6^ PFU of HSV-1 in 10 μL was applied intranasally. At 24 and 48 h post infection, mice were treated intranasally with 20 μL of 0.9% NaCl solution containing 20 μg /mL of selected nanoparticles. The dose of NPs were selected on the basis of previous works, which used the dose of no more than 10 μg/mL since higher doses caused acanthosis of epithelial tissues (see - supplementary materials). 15–17 Mice were monitored daily and scored as described in.^14^ At day 3 or 7 p. i., mice were sacrificed by cervical dislocation and tissues (brains, trigeminal ganglia, and nasal cavities) were either stored in fix RNA (Eurx) or used to prepare single cell suspensions.

### Flow Cytometry Analysis

Single cell suspensions were prepared as described in.^14^ To avoid unspecific staining, the Fc receptor block-rat anti-CD16/32 antibody (2.4G2) (BD Biosciences) was used according to the manufacturer’s protocol. The detailed list of antibodies used to detect T cells, NK cells, microglia, monocytes, Langerhans cells is given in Table S1 (supplementary materials). HSV-1 specific T cells were detected using the SSIEFARL-PE tetramer (Creative Biolabs, Shirley, NY, USA) Stained cells were acquired using CytoFLEX LX (Beckman Coulter) and analyzed using FlowJo software (Tree Star, Ashland, OR, USA).

### Statistics

For statistical analysis, GraphPad Prism version 7 (GraphPad software) was used. To compare the differences between the groups, the Mann–Whitney *U*-test and Wilcoxon test, were used and the results are shown as mean ± standard error of the mean (SEM). The p < 0.05 was considered statistically significant.

## Results

### Characterization of Nanoparticles

**Figure 1:**
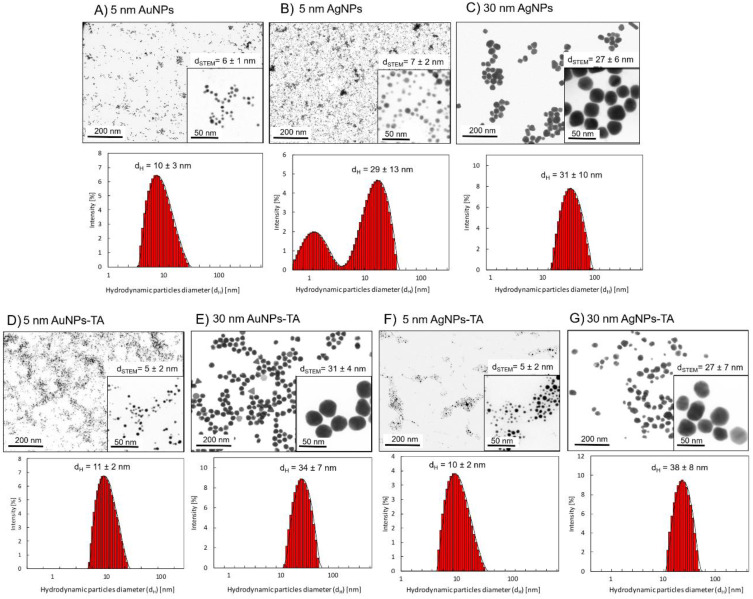
HR-STEM images and DLS size distribution histograms of the mNPs: citrate-(**A-C**) and tannic acid-modified (**D-G**).

Metallic nanoparticles were characterized with DLS (hydrodynamic size d_H_ and colloidal stability) and HR-STEM (metallic core size - d_STEM_, size distribution, and shape) techniques. The summary of characterization results for mNPs are presented in Figure 1A–G. For citrate-stabilized mNPs, the sizes of the metallic cores were as follows: d_STEM_ = 6 ± 1 nm (5 nm AuNPs); d_STEM_ = 7 ± 2 nm (5 nm AgNPs); d_STEM_ = 27 ± 6 nm (30 nm AgNPs). The hydrodynamic size of the mNPs was equal: d_H_ = 10 ± 3 nm (5 nm AuNPs), d_H_ = 29 ± 13 nm (5 nm AgNPs), and d_H_ = 31 ± 10 nm (30 nm AgNPs) (Figure 1A–C). For TA-modified mNPs, the sizes of the metallic cores were as follows: d_STEM_ = 5 ± 2 nm (5 nm TA-AuNPs); d_STEM_ = 31 ± 4 nm (30 nm TA-AuNPs); d_STEM_ = 5 ± 2 nm (5 nm TA-AgNPs); d_STEM_ = 27 ± 7 nm (30 nm TA-AgNPs). The hydrodynamic size of mNPs-TA was equal to d_H_ = 11 ± 2 nm (5 nm TA-AuNPs), d_H_ = 34 ± 7 nm (30 nm TA-AuNPs), d_H_ = 10 ± 2 nm (5 nm TA-AgNPs), and d_H_ = 38 ± 8 nm (30 nm TA-AgNPs) (Figure 1D–G). The key storage parameters that can potentially affect the stability of silver nanoparticles are: sunlight, access to oxygen and storage time. We studied the effect of all those parameters on the colloidal stability of TA-AgNPs and did not observe their significant impact on the stability of TA-AgNPs (stability investigated using the DLS technique enabling the monitoring of colloidal stability and hydrodynamic diameter of particles). Stability tests of TA-AgNPs conducted in transparent vials with access of sunlight (Supplementary Figure 4a) did not show any changes in the hydrodynamic diameter during the 35 days of the experiment. The obtained results are comparable to those obtained for the colloid stored in a dark vial (Supplementary Figure 4b) and the colloid stored in a darkness (Supplementary Figure 4c). Similarly, storage of TA-AgNPs colloid in conditions with full access to oxygen form the air did not affect the colloidal stability of the nanoparticles during 35 days of studies (Supplementary Figure 4d). Studies conducted over time (up to 35 days) demonstrate long-term stability of TA-AgNPs colloid.

### Direct Antiviral Mechanism of TA-Modified Nanoparticles is Size Dependent

**Figure 2:**
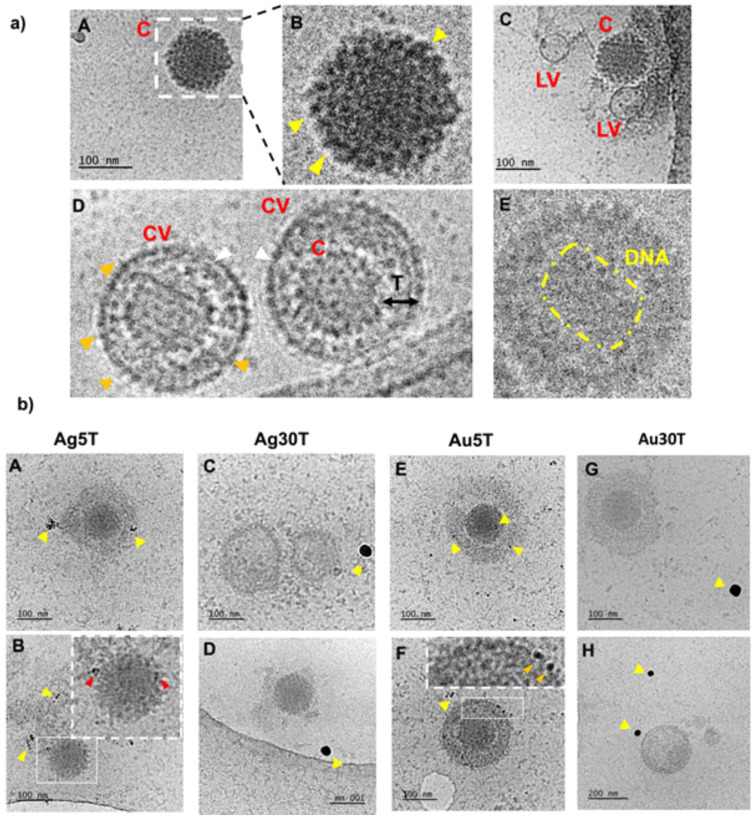
Cryo-TEM imaging of HSV-1 treated with nanoparticles. The HSV-1 virus was treated with nanoparticles for 5 minutes at room temperature (RT), followed by preparation for Cryo-TEM imaging as described in the Materials and Methods section. Panel (**a**) illustrates the structure of HSV-1 in the absence of nanoparticle treatment. Various morphological forms of HSV-1 were observed - icosahedral capsids (A–C) were identified, with yellow arrows indicating the presence of spike proteins on the capsid surface. Additionally, empty virions (C) were noted, along with complete virions containing an envelope, capsid, and tegument (D). White arrows highlight the viral envelope, while Orange arrows indicate envelope-associated proteins. In some instances, the genetic material of the virus was visible (E). Panel (**b**) shows HSV-1 cells treated with nanoparticles (A and B) Ag5T; (C and D) Ag30T; (E and F) Au5T; (G and H) Au30T). Yellow arrows indicate the nanoparticles under investigation, whereas red and orange arrows highlight their interactions with the structural elements of the virus. Red arrows indicate interactions with the capsid and orange arrows indicate interactions with the viral envelope.

**Abbreviations**: C, capsid; LV, lacking virions; CV, complete virion; T, tegument.

First, we decided to elucidate the previously observed mechanisms of HSV-1–NPs interactions that lead to virus inactivation using cryo-TEM (Figure 2). For this purpose, NPs were incubated with the HSV-1 virus for 5 min and then visualized using cryo-TEM. The control panel (Figure 2A) highlighted the diverse morphological forms of the HSV-1 virus. Complete HSV-1 virions with icosahedral capsids and viral envelopes were clearly visible with spike proteins identifiable on the capsid surface. Additionally, empty viral forms were observed, characterized by the presence of the viral envelope without the capsid (Figure 2A and C). In the samples treated with nanoparticles (Figure 2B), structural changes in HSV-1 virions were evident. Application of 5 nm TA-AgNPs resulted in the destruction of the viral envelope, accompanied by noticeable interactions between the nanoparticles and both disrupted envelope and capsid proteins (Figure 2A and B). For larger 30 nm TA-AgNPs, no clear interaction with HSV-1 virions was observed; however, destroyed viral envelopes were detected, with nanoparticles visible loosely connected with remains of the virions.

In contrast, small 5 nm TA-AuNPs exhibited visible interactions with the viral envelope and HSV-1 capsid (Figure 2B, E and F). Meanwhile, larger 30 nm TA-AuNPs did not demonstrate any significant interaction with the structural elements of the virus (Figure 2B, G and H).

### Antiviral Effects of TA-Modified Nanoparticles in vitro are Size and Metal-Dependent

**Figure 3:**
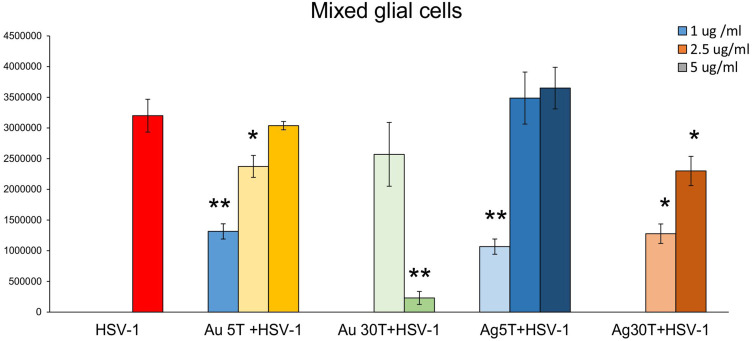
The dose of tannic-acid modified Ag/AuNPs (TA- Ag/AuNPs) necessary to inhibit HSV-1 infection of glial cells is size and metal-dependent. The mixed glial cells were infected for 6 h, then washed and treated with a range of tannic acid modified AgNPs or AuNPs sized 5 and 30 nm concentrations (1–5 µg/mL). HSV-1 copies titration by qPCR was performed at 24 h p.i. Data from three independent experiments are presented as mean ± SEM. *Represents significant differences with p ≤ 0.05, **p ≤ 0.01 in comparison to untreated infected control (two-way ANOVA test).

Next, we performed antiviral tests in vitro to correlate the obtained images with the outcomes of in vitro HSV-1 infection. Based on our previous results, which showed that the toxicity of TA-modified nanoparticles is size- and cell-dependent,^17^ we chose the same nanoparticle concentrations of 2.5 µg/mL for neuronal and glial cells and 5 µg/mL for Vero cells. First, we used Vero cells to study the antiviral activity of tannic acid modified 5 and 30 nm Au/AgNPs, with the classical plaque reduction assay (Supplementary Figure 1A). The tested tannic acid-modified NPs demonstrated very good antiviral activity (p ≤ 0.01) except for 30 nm AuNPs, for which we observed decreased viral titers; however, the results were not statistically significant (Supplementary Figure 1A). Furthermore, we tested the effects of post-infection treatment with NPs applied immediately before virus egress from cells (6 h post infection). We found that application of NPs to infected cells was efficient only for Neuro2a cells (p ≤ 0.05), except for 5 nm TA-AgNPs and mixed glial cells treated with 5 and 30 nm TA-AgNPs (p ≤ 0.05) (Supplementary Figure 1B). However, the antiviral effect observed for the treatment of mixed glial cells was size- and dose-dependent, showing that we were able to determine an effective dose for each type of NP (Figure 3). We applied non-toxic concentrations, as tested previously by Janicka et al 2022,^17^ and found that while small TA-AuNPs exerted virucidal activity at 1 and 2.5 μg/mL (p = 0.049), 30 nm TA-AuNPs were efficient as antivirals at only 5 μg/mL (p = 0.001) (Figure 3). For AgNPs, small 5 nm NPs were effective only at 1 μg/mL, whereas 30 nm TA-AgNPs were effective at both 2.5 and 5 μg/mL, (p ≤ 0.05) (Figure 3).

### Cells of Monocyte Origin Internalize Nanoparticles and Become Activated

**Figure 4:**
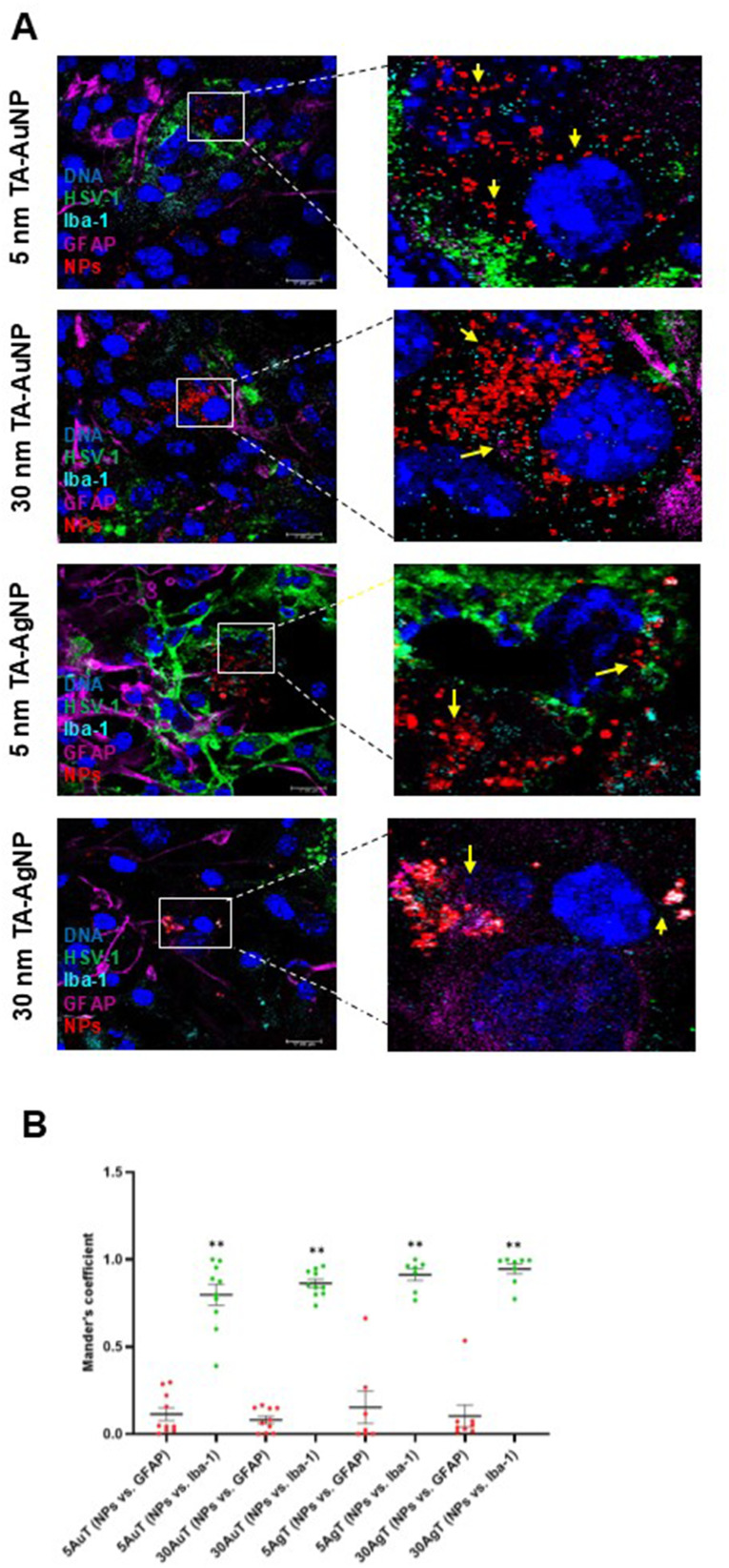
TA-Au/AgNPs are internalized by microglia. The mixed glial culture were plated on slides and infected for 6 h with HSV-1, then washed and treated with a range of tannic acid modified AgNPs, or AuNPs sized 5 and 30 nm. (**A**) Twenty-four hours post infection, the fixed cells were stained for microglia (Iba-1+, turquoise), astrocytes (GFAP+, purple), HSV-1 (green), counterstained with DAPI for DNA (blue) and analyzed in confocal microscope. Nanoparticles were visualized in a reflection mode (red). Yellow arrows point to nanoparticles. (**B**) The overlap of nanoparticle signal with GFAP- or Iba-1-positive cells calculated as Mander’s Colocalization Coefficients. Data from three independent experiments are presented as mean ± SEM. Two-way ANOVA test p ≤ 0.01 **.

Considering the observed antiviral effects in mixed glial cells and previously shown internalization of silver and gold nanoparticles,^17^ we used confocal microscopy to identify and quantify the co-localization of NPs with microglia or astrocytes in HSV-1-infected mixed glial primary cultures (Figure 4). Mixed glial cultures were infected with HSV-1 for 6h and then treated with 5 nm TA-Au/AgNPs or 30 nm TA-Au/AgNPs at non-toxic dose. After 24 h from infection, cultures were fixed and stained for microglia (IBA-1+) and astrocytes (GFAP+) using immunofluorescent antibodies. The presence of NPs was detected in the laser reflection mode (Figure 4A). Mander’s Colocalization Coefficient of 1 for the nanoparticle channel with regard to the Iba-1 channel indicated 100% presence of the nanoparticles within microglia (Figure 4B). Co-localization of NPs with microglia was observed for all tested NPs (Figure 4B).

**Figure 5:**
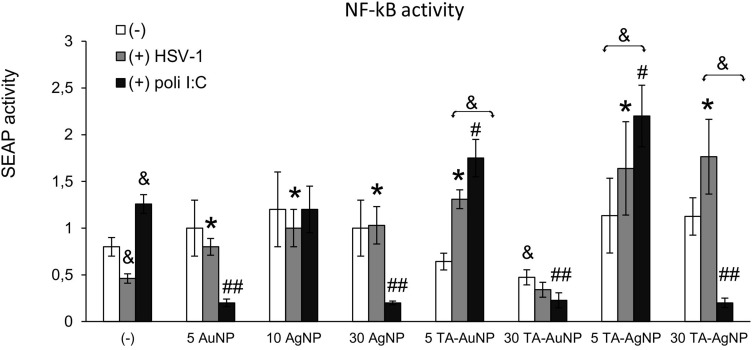
Tannic acid modified NPs significantly activate NF-κB activity in HSV-1- infected RAW-Dual™ cells. The RAW cells were uninfected, infected with HSV-1 (1 PFU/cell) or treated with poly (I:C) at 1 μg/mL for 2 h, then tannic acid-modified or unmodified 5 nm AgNPs, 30 nm AgNPs, 5 nm AuNPs and 30 nm AuNPs were added. After 6 h, the activation of NF-κB pathway was measured as SEAP activity. Data from three independent experiments are presented as mean ± SEM. Two-way ANOVA test. *Represents p ≤ 0.05 in comparison to HSV-1-infected control, ^&^p ≤ 0.05 in comparison to uninfected control, and p ^#^≤ 0.05, ^##^p ≤ 0.01 in comparison to poly (I:C) treated control.

Microglia are of monocyte origin, and similar to monocytes present in other body parts, microglia are responsible for mounting of the innate immune response. To determine whether internalization of NPs by HSV-1-infected monocytes had any influence on their activation as innate immune cells, we used RAW-Dual™ cells with activity reporting the activation of NF-κB intracellular pathways related to inflammatory and antiviral responses (Figure 5). The addition of non-toxic doses of TA-modified and unmodified AgNPs or AuNPs did not significantly increase NF-κB activity in RAW-Dual™ cells after 6 h of treatment, except for 30 nm TA-AuNPs, which significantly reduced NF-κB activity (p = 0.041) (Figure 5). At 6 h p.i., NF-κB activity was significantly lower compared to uninfected control (p = 0.021) (Figure 5). Unmodified 5 nm AuNPs, 10 nm AgNPs and 30 nm AgNPs increased the levels of NF-κB activity in HSV-1-infected RAW-Dual™ cells to those observed for uninfected control (p ≤ 0.05) (Figure 5). Tannic acid-modified 5 nm AuNPs, 5 nm AgNPs and 30 nm AgNPs increased NF-κB activity in HSV-1- infected RAW-Dual™ monocytes more significantly than unmodified NPs (p ≤ 0.05) (Figure 5). However, the addition of 30 nm TA-AuNPs showed no influence upon NF-κB activity in HSV-1 infected RAW-Dual™ monocytes (p = 0.08) (Figure 5). We further used polyinosinic-polycytidylic acid [poly(I:C)], a synthetic analog of double-stranded RNA (dsRNA), recognized by TLR3 receptors. Interestingly, the addition of 5 nm AuNPs and 30 nm AgNPs reduced poly(I:C)-induced NF-κB activity, whereas for TA-modified Ag/AuNPs, the observed effects were size-dependent (Figure 5). Small, 5 nm TA-AuNPs or TA-AgNPs significantly upregulated NF-κB activity, but a significant decrease was detected for 30 nm TA-AuNPs or TA-AgNPs (p ≤ 0.05) (Figure 5).

The mixed glial cells consisted of microglia and astrocytes in a ratio of 2:8. Therefore, we expect that mixed glial cultures can react by activating both HSV-1 infection and NPs internalization. Indeed, HSV-1 infected mixed glial cultures treated 6 h post infection with TA-modified NPs of different sizes and metal types demonstrated a significant increase in mRNA expression levels for IFN-α, CXCL9, and CXCL10 compared to untreated HSV-1-infected mixed glial cultures (p ≤ 0.05) (Supplementary Figure 2).

### Only TA-Modified Nanoparticles Decrease HSV-1 Titers During in vivo Infection

**Figure 6:**
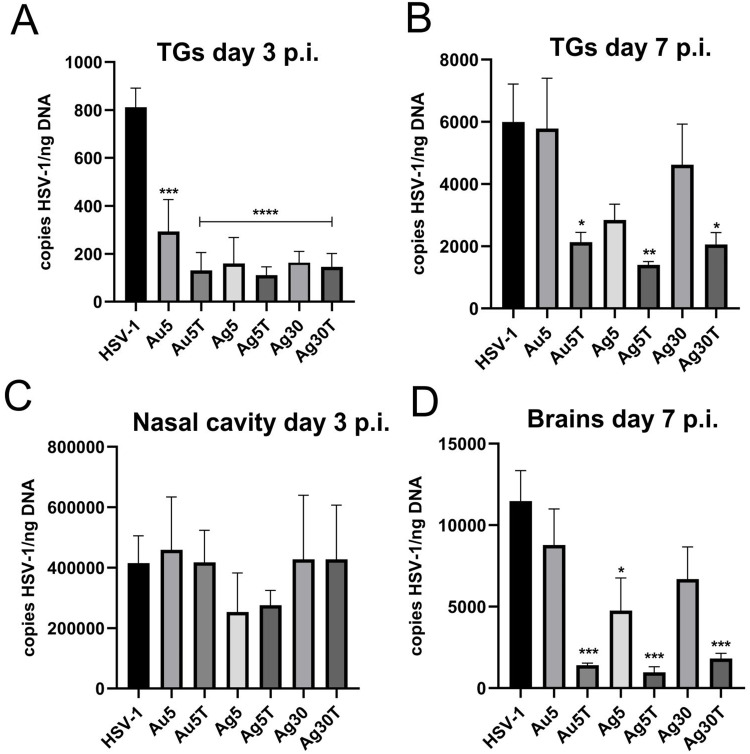
Treatment with tannic acid-modified Ag/AuNPs of both 5 and 30 nm reduces HSV-1 infection in vivo much better than with unmodified NPs. C57BL/6 mice infected intranasally with HSV-1 were treated two times every 24 h with tannic acid-modified or unmodified 5 nm AgNPs, 30 nm AgNPs, 5 nm AuNPs or 0.9% NaCl (control). Trigeminal ganglia (TG), nasal cavities and brains were collected at 3 (**A and C**) and 7 days p.i. (**B and D**), and subjected to measurement of HSV-1 gB titers (copies/ng DNA) by qPCR (N = 10). The bars represent means ± SEM. * represents significant differences with p ≤ 0.05, **p ≤ 0.01, ***p ≤ 0.001 and extremely significant at p ≤ 0.0001 **** in comparison to untreated infected tissues.

Taking into account the results of the in vitro studies, we further investigated whether local treatment of mucosal tissues with TA-modified Ag/AuNPs can help to limit HSV-1 infection. For this purpose, we applied 0.4 μg/mouse of 5 nm AuNPs, 5 nm TA-AuNPs, 5 nm AgNPs, 5 nm TA-AgNPs, 30 nm AgNPs and 30 nm TA-AgNPs at 24 and 48 h post infection (p.i). (Figure 6). Control mice were treated with 0.9% NaCl. HSV-1 titers were measured in nasal cavities (3 d p.i)., trigeminal ganglia (TGs) (3 and 7 days p.i)., and brains (7 d p.i). We found that intranasal treatment with TA-modified and unmodified Ag- or AuNPs led to significantly lower titers of HSV-1 in TGs at 3 days p.i. (p ≤ 0.001) (Figure 6A), but not in the nasal cavities (Figure 6C). At 7 day of infection, a significant decrease in HSV-1 titers was detected only in TGs and brains of mice treated with TA-modified 5 nm AgNPs, 5 nm AuNPs and 30 nm AgNPs (p ≤ 0.05) (Figure 6B and D, respectively).

### TA-Modified Nanoparticles Provide Better Antiviral Response During HSV-1 in vivo

**Figure 7:**
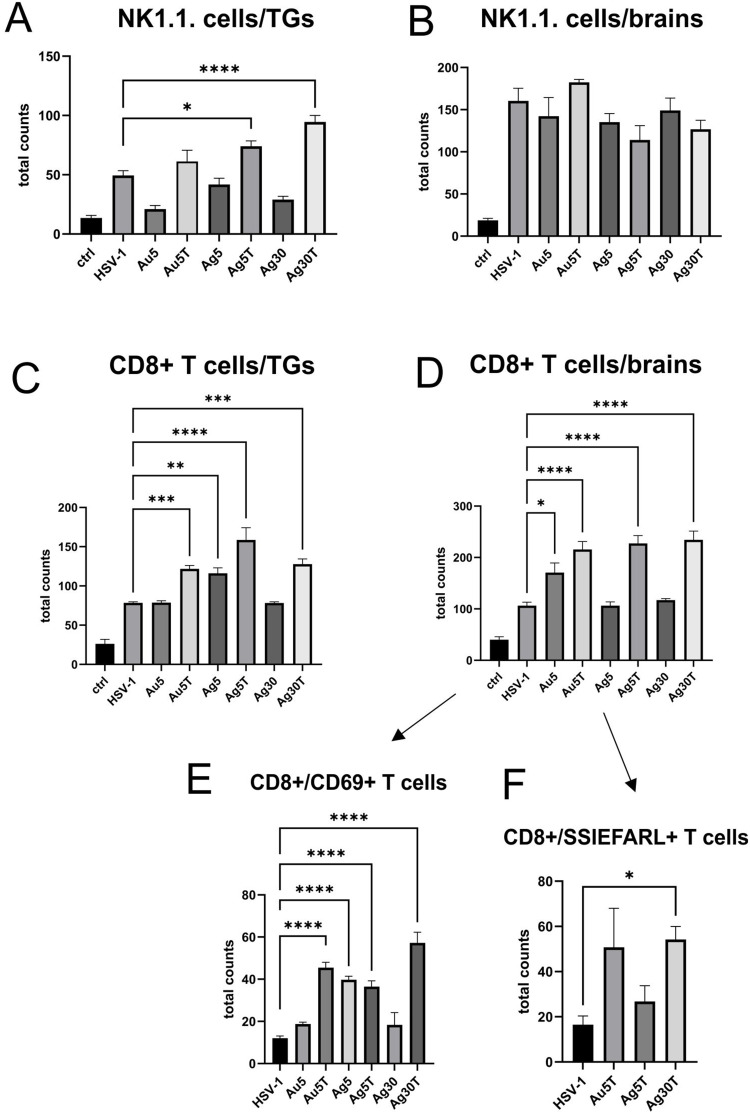
Tannic acid-modified Ag/AuNPs of both 5 and 30 nm activate early antiviral response in HSV-1 infection much better than with unmodified NPs. Total counts of (**A and B**) NK cells, (**C and D**) CD8+ T cells, (**E**) CD8+CD69+ T cells and (**F**) CD8+SSIEFARL+ T cells isolated from TGs and brains of HSV-1-infected mice at 7 days p.i., and treated with tannic acid-modified or unmodified 5 nm AgNPs, 5 nm AuNPs or 0.9% NaCl. Results are expressed as mean ± SEM for N = 7. *Represents significant differences with p ≤ 0.05, **p ≤ 0.01, ***p ≤ 0.001 and extremely significant at p ≤ 0.0001 **** in comparison to untreated infected tissues.

To understand how tannic acid modification of Ag/AuNPs can facilitate mounting of the early anti-HSV-1 immune response, we subjected the cell suspensions prepared from the trigeminal ganglia (TGs) and whole brains at 7 d p.i. to immunophenotyping for CD4+ T cells, CD8+ T cells, and NK cells, (Figure 7). At 7 d p.i., TGs, but not brains, from mice treated with 5 nm and 30 nm TA-AgNPs demonstrated significant upregulation of NK cells (p ≤ 0.05) (Figure 7A and B). Application of 5 nm TA-AuNPs upregulated the numbers of NK cells, but unsignificantly (p = 0.59) (Figure 7A). Treatment with unmodified Ag/AuNPs of both sizes did not lead to significant activation of NKs compared with the untreated control (Figure 7A). Furthermore, treatment with TA-modified 5 nm Ag/AuNPs or TA-modified 30 nm AgNPs resulted in significantly upregulated infiltration of CD8+ T cells into the TGs and brains in comparison to the untreated control (p ≤ 0.01) (Figure 7C and D). Interestingly, we found that only unmodified 5 nm AgNPs in TGs and unmodified 5 nm AuNPs in brains significantly induced the numbers of CD8+ T cells (p ≤ 0.05) (Figure 7C and D). Additionally, we found that post-infection treatment with tannic acid-modified Ag/AuNPs of both sizes led to significant infiltration of activated CD8+CD69+ T cells and cytotoxic CD8+ T cells recognizing HSV-specific SSIEFARL antigens (p ≤ 0.05) (Figure 7E and F).

We also found that the expression of IFN-gamma, CXCL9, and CXCL10, known for their antiviral activity, increased in the organs of mice treated with tannic acid-modified nanoparticles (Supplementary Figure 3). We detected significantly higher expression of IFN-gamma mRNAs in the TGs of mice treated with 5 nm TA-AuNPs, 5 nm TA-AgNPs and 30 nm TA-AgNPs both at 3 and 7 d p.i. compared to unmodified NPs (p ≤ 0.05) (Supplementary Figure 3A and B), whereas only TA-AgNPs induced IFN-γ mRNA expression in the brain at 7 d p.i. (p ≤ 0.05) (Supplementary Figure 3C). TA-AgNPs induced the expression of CXCL9 mRNA in all tested tissues and times p.i. (p ≤ 0.05) (Supplementary Figure 3), except for 5 nm TA-AuNPs in TGs at 3 d p.i. Expression of CXCL10 mRNA was upregulated by tannic-modified NPs only in TGs at both times p.i. and by 30 nm TA-AgNPs in the brain (p ≤ 0.05) (Supplementary Figure 3C).

### TA-Modified Nanoparticles Modulate Inflammatory Response During HSV-1 in vivo Infection

**Figure 8:**
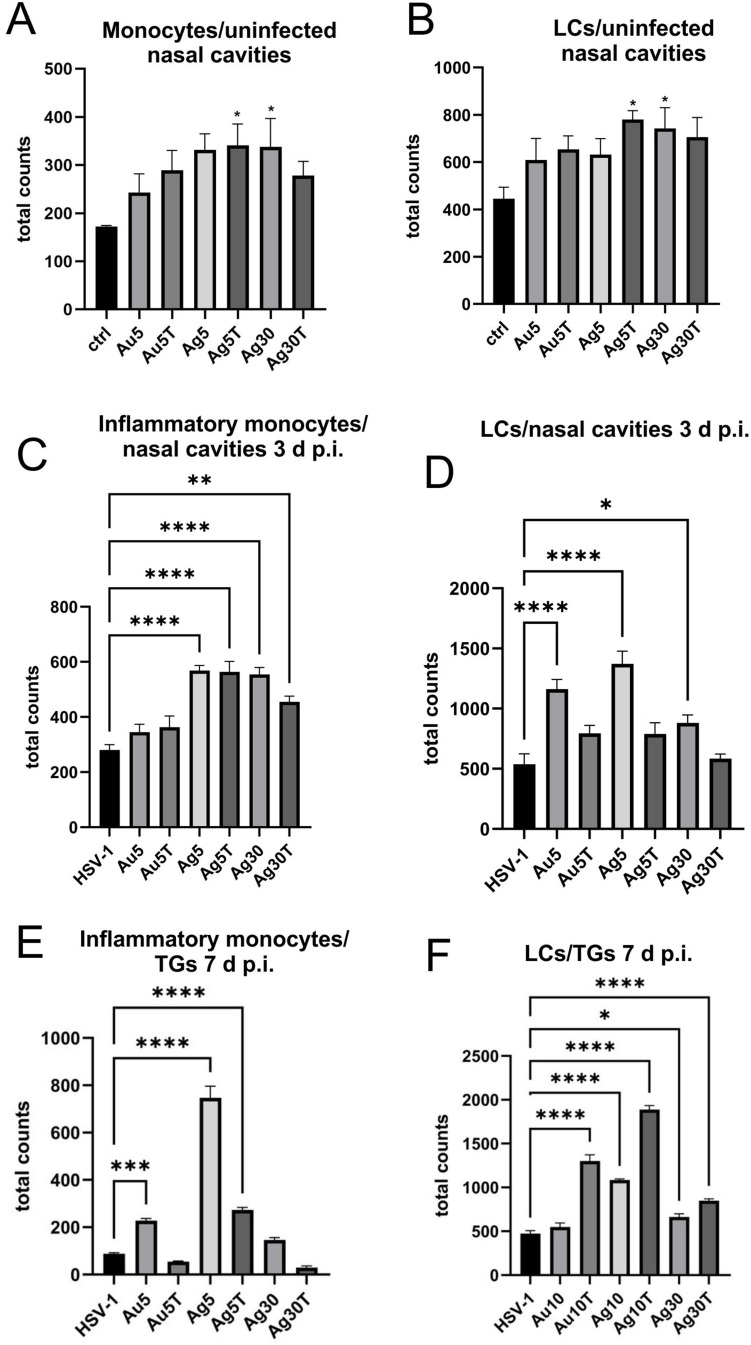
Tannic acid-modified Ag/AuNPs of both 5 and 30 nm modulate local inflammatory and early antiviral response in HSV-1 infection. Total counts of (**A**) monocytes, (**C** and **E**) inflammatory monocytes and (**B, D, F**) Langerhans cells (LCs) isolated from nasal cavity, trigeminal ganglia (TGs) and brains of uninfected and HSV-1-infected mice at 3 or 7 days p.i., and treated with tannic acid-modified or unmodified 5 nm AgNPs, 30 nm AgNPs, 5 nm AuNPs or 0.9% NaCl. Results are expressed as mean ± SEM for N = 7. *Represents significant differences with p ≤ 0.05, **p ≤ 0.01, ***p ≤ 0.001 and extremely significant at p ≤ 0.0001 **** in comparison to HSV-1 infected tissues.

Considering the activation of monocytes observed in vitro, we examined the numbers of monocytes and Langerhans cells in cell suspensions prepared from the nasal cavities of uninfected mice treated with NPs. (Figure 8A and B). We found that all NPs, irrespective of their size, metal, or tannic acid modification, upregulated the numbers of monocytes and Langerhans cells (LCs) infiltrating to nasal mucosa (Figure 8A and B), but not of NK cells (data not shown). However, only 5 nm TA-AgNPs and 30 nm AgNPs induced significant infiltration in control, uninfected mice (p ≤ 0.05) (Figure 8A and B). Because local inflammation at the site of HSV-1 infection attracts homing of Langerhans cells, which are responsible for carrying HSV-1 antigens to the local lymph nodes, we further examined the infiltration of monocytes and LCs at the local mucosa of the nasal cavity in HSV-1-infected mice treated with all types of NPs at 3 d p.i. (Figure 8C and D). We observed that treatment with AgNPs (modified and unmodified) at 5 and 30 nm induced significant infiltration of inflammatory monocytes compared with untreated infected mice (p ≤ 0.05) (Figure 8C). No significant upregulation of infiltration of inflammatory monocytes into infected mucosal tissues was observed in the AuNP-treated mice (Figure 8C). Furthermore, treatment with unmodified 5 nm AuNPs, 5 and 30 nm AgNPs led to a significant infiltration of Langerhans cells within the nasal mucosa at 3 d p.i. (Figure 8D). HSV-1 reaches TGs–2-3 days after infection, establishes latency, and becomes a target of immune-competent cells.

At 7 d p.i., TGs isolated from mice treated with unmodified 5 nm Ag/AuNPs and 30 nm AgNPs showed significantly increased counts of inflammatory M1 monocytes compared with untreated infectious controls (p ≤ 0.05) (Figure 8E). However, an opposite effect was observed for LCs in mice treated with TA-modified 5 nm Ag/AuNPs and 30 nm AgNPs –TGs from these mice showed significantly increased numbers of LCs identified in cell suspensions (p ≤ 0.05) (Figure 8F).

**Figure 9:**
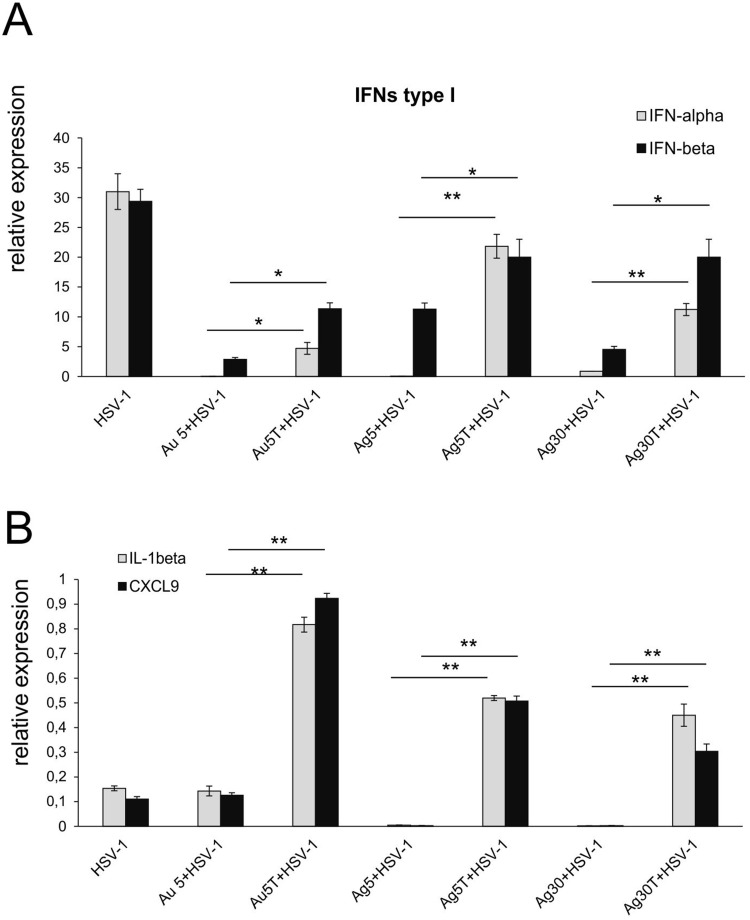
Tannic acid-modified Ag/AuNPs of both 5 and 30 nm modulate mucosal inflammatory response in HSV-1 infection better than unmodified NPs. Cytokine and chemokine expression in the nasal cavities at 3 days p.i. after treatment with tannic acid-modified or unmodified 5 nm AgNPs, 30 nm AgNPs, 5 nm AuNPs or 0.9% NaCl. Levels of IFN-α, IFN-β (**A**) as well as CXCL1 and CXCL9 (**B**) mRNAs are shown as expression relative to control based on the 2-∆∆Ct method. N = 7. *Represents significant differences with p ≤ 0.05, **p ≤ 0.01 in comparison to uninfected tissues.

Increased infiltration of immune-competent cells within the nasal cavity was followed by an advantageous profile of cytokines and chemokines important for development of an early immune response (Figure 9). Surprisingly, while treatment with unmodified nanoparticles led to a significant decrease in the interferon type I response (IFN-α and IFN-β) compared to HSV-1 infected untreated mice (p ≤ 0.001) (Figure 9A), the use of TA-modified NPs helped to regain the expression of IFN-α and IFN-β mRNAs (Figure 9A). This effect was particularly observed for TA-AgNPs sized 5 and 30 nm and IFN-β mRNA expression compared to the same size, unmodified AgNPs (p ≤ 0.05) (Figure 9A), where the levels of IFN-α did not differ from those observed in infected but untreated nasal mucosa (Figure 9A). To a lesser extent, all tested TA-modified Ag/AuNPs significantly upregulated IFN-α mRNA levels compared with unmodified NPs (p ≤ 0.05) (Figure 9A). Furthermore, all nasal mucosa from infected mice treated with TA-modified NPs, irrespective of size and metal type, showed significant upregulation of CXCL9 and IL-1β mRNA expression compared to unmodified NPs (p ≤ 0.01) (Figure 9B).

**Figure 10:**
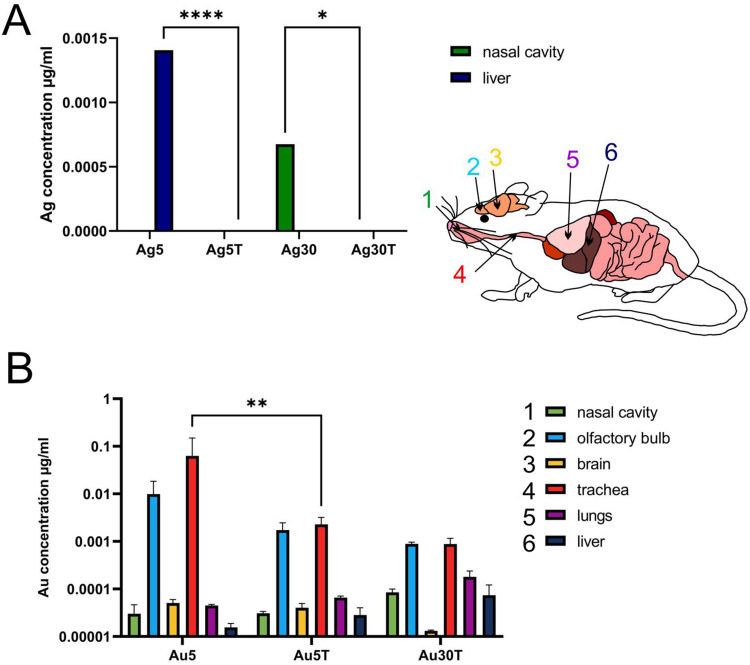
Tannic acid-modified and unmodified Ag/AuNPs of both sizes - 5 and 30 nm accumulate in tissues differently. C57BL/6 mice were treated intranasally two times every 24 h with tannic acid-modified or unmodified 5 nm AgNPs, 30 nm AgNPs, 5 nm AuNPs or 30 nm TA-AuNPs. Nasal cavities, brains with olfactory bulbs, trachea, lungs and livers were collected at 2 days post-treatment and subjected to inductively coupled plasma mass spectrometry (ICP-MS) for Au (**A**) and Ag (**B**) content in extracts prepared from collected organs. Data from three independent experiments are presented as mean ± SEM. Two-way ANOVA test with p ≤ 0.05 *, p ≤ 0.01 **, and extremely significant at p ≤ 0.0001 **** in comparison to unmodified NPs (TA-AuNPs vs AuNPs, etc.).

Since the topical application of metal nanoparticles leads to their internalization by phagocytizing cells, such as monocytes and macrophages, we also checked for the presence of Ag and Au in tissue extracts prepared from nasal cavities, tracheas, lungs, olfactory bulbs, brains (the remaining part, including cortex, cerebellum, and medulla), and liver of mice treated post-infection with AgNPs or AuNPs. We found that it was possible to detect silver only in the livers (5 nm AgNPs) and nasal cavities (30 nm AgNPs) at 7 d p.i. (Figure 10A), and modification with tannic acid led to significantly lower accumulation of Ag in these tissues (p ≤ 0.05) (Figure 10A). In contrast, we detected Au in all the tested tissues (Figure 10B). Lower Au concentrations were observed after treatment with tannic acid modified 5 nm AuNPs in the trachea (p = 0.01) and in olfactory bulbs (insignificant) compared to unmodified 5 nm AuNPs (Figure 10B). The highest amounts of Au were found in the nasal cavity, olfactory bulbs, lungs, and liver (Figure 10B).

## Discussion

The present study demonstrated the role of the interaction between size, material, and surface modification, and the antiviral activity of NPs against HSV-1 infection in vitro and in vivo.

Antiviral activities of metal NPs, such as Au- and Ag-based systems, have been widely studied and described.^1^,^2^,^4^ However, as other authors have suggested, depending on the virus size and shape of the outer structure (enveloped vs non-enveloped, protein spikes, etc)., NPs of different composition, modification and sizes may exert efficient virucidal activity. Synthesis methods often lead to the presence of different organic compounds that stabilize NPs, whose function is to coat the surface and avoid aggregation, leading to a loss of characteristic properties.^4^,^5^ Sometimes, coating is performed to mimic heparan sulfate (HS) and to increase virucidal effects.^21^,^22^ We have previously demonstrated that TA-AgNPs of 13, 33, and 46 nm can effectively inhibit HSV-2 infection both in vitro and in vivo.^14–18^ We also showed that antiviral action requires direct interaction with the virion surface, and this interaction requires the presence of tannic acid (TA) on the AgNPs.^14–18^ The role of tannins in blocking interactions between cell-surface glycosaminoglycans and HSV-1 glycoproteins has been shown previously, also for other viruses that use glycosaminoglycans for entry, such as human cytomegalovirus (HCMV), hepatitis C virus (HCV), dengue virus (DENV), measles virus (MV), and respiratory syncytial virus (RSV).^23^,^24^ Tannins interact with specific sites on the viral glycoproteins involved in attachment, membrane fusion, or cell spread, and further compete with cell-surface GAGs for the virus particles.^22^,^23^

To understand how the size, composition, and tannic acid presence influence the antiviral properties of nanoparticles, we compared the antiviral activity of TA-modified metal NPs of two sizes (5 and 30 nm) and prepared of Ag and Au. Using direct antiviral tests (virus-nanoparticle interaction), we found that small-sized TA-modified Ag and Au nanoparticles showed similarly efficient antiviral activity against HSV-1, while 30 nm TA- AuNPs showed very weak antiviral activity in contrast to TA-30 nm AgNPs. We previously demonstrated that upon interaction with 30 nm AgNPs, HSV-2 virions interact with several NPs; however, this imaging was performed in fixed samples.^16^ By using cryo-TEM technique, we were able to observe immediate virus-nanoparticle interactions. We found that small-sized TA-modified Ag/Au nanoparticles (5 nm) interacted in a lasting manner with the envelope, forming the outer structure of the HSV-1 virion. TA-modified small NPs remained bound to the envelope, although the virus envelope was partially destroyed (Figure 2). The tannic acid is responsible for these interactions, as we previously showed that only TA-modified NPs can interact with HSV-2 and form aggregates with the virus envelope.^25^ Since HSV-1 differs from HSV-2 only in the structure of surface glycoproteins, the mechanism of TA-interaction for both viruses should be similar.^26^ Concerning the larger Au/AgNPs we did not find images of stable, long-lasting interactions of 30 nm AuNPs with the virions, while interaction with 30 nm TA-AgNPs led to destruction of the virions – its envelope was “shed off” and even the tegument becomes damaged. Therefore, we can conclude that the interaction between TA-NPs and HSV-1 virions depends on the NPs size and composition, with smaller sizes being independent of the NPs composition, whereas for larger NPs, the metal type is important for HSV-1 inhibition. Vonnemann et al^27^,^28^ revealed that surface-area-normalized polysulfated gold nanoparticles >50 nm more efficiently inhibited the binding of vesicular stomatitis virus (VSV) to cells than did smaller particles. Inhibition of viral infection by larger nanoparticles was up to two orders of magnitude more efficient than that by smaller particles, suggesting different mechanisms of virus inhibition. The authors concluded that larger AuNPs acted as efficient cross-linkers between virions, whereas smaller AuNPs decorated the surface of virions in a manner similar to our data.^27^,^28^ We suggest that tannic acid in our study may act as a competitive binding inhibitor, helping to bind virions and inactivate them, possibly by acting as a cross-linker, allowing the formation of aggregates of nanoparticles with the virions.

Furthermore, the size of nanoparticle plays an important role in determining the process of cellular uptake and toxicity. It has been shown that NPs of smaller sizes ranging from 10 to 30 nm may actively cross the cell membrane, whereas uptake of NPs of larger sizes requires endocytosis, consisting a passive mechanism.^29^ Our previous studies also showed that TA-Ag/AuNPs of different sizes were internalized by both monocytes and dendritic cells^15^,^17^ and the inhibitor of clathrin-mediated endocytosis blocked the uptake of TA-Ag/AuNPs by dendritic cells.^17^ Both in monocytes and dendritic cells, NPs were localized mainly within electron-dense vacuoles attached to the inner vacuole membrane.^15^,^17^ Furthermore, cytochalasin D, an inhibitor of phagocytosis and micropinocytosis, decreased the internalization of TA-Ag/AuNPs.^17^

Microglia are resident macrophages of the CNS, which play a crucial role in maintaining brain homeostasis but also in the protection of the brain from invading pathogens, synaptic plasticity, neuronal repair, and neurogenesis.^30^,^31^ Upon HSV-1 infection, microglial cells undergo abortive infection, which further induces a burst of pro-inflammatory cytokine and chemokine production, activating other glial cells within the CNS and helping to mount the adaptive immune response in the CNS.^32^,^33^ Here, we found that microglia internalize NPs. Furthermore, the application of TA-Ag/AuNPs to treat HSV-1 infection provides a better antiviral response, measured by production of IFN-α, CXCL9, and CXCL10, which in turn attract T and NK cells to infiltrate HSV-infected sites.^34^

It has been shown that HSV infection of monocytes/macrophages results in totally interrupted or partially restricted replication (non-productive or abortive infection).^35^ Activation of NF-κB during HSV-1 infection plays a central role in restriction of infection in monocytic cells, preserving these cells from both viral replication and apoptosis.^35^,^36^ The use of NF-κB inhibitors enhances viral replication in HSV-1 infected monocytic cells.^37^ Here, we found that the addition of both silver and gold nanoparticles to HSV-1 infected monocytes helped to induce NF-κB activity to the level observed in control cells (unmodified Ag/AuNPs) or even higher levels upon treatment with TA-Ag/AuNPs. However, the application of large, 30 nm TA-AuNPs decreased NF-κB activity in monocytes upon HSV-1 infection. The activation of NF-κB in HSV infection is cell type-dependent and involves different pathways.^37^,^38^ The use of the TLR3 ligand poly (I:C) selectively activates numerous signaling pathways, further resulting in the production of type I IFNs and NF-B activation-dependent production of TNF-α, IL-6, and CXCL10.^39^,^40^ In our study, activation of NF-κB activity induced by poly (I:C) was modulated by Ag/AuNPs depending on the size, metal type, and presence of tannic acid modification, with a clear downregulation of the TLR3-dependent pathway by large Au/AgNPs, irrespective of the metal type. Duffy et al (2018) previously demonstrated that at non-toxic dose, 20 nm AgNPs may induce microglial inflammatory response through a NF-κB mediated pathway.^41^ Other authors using the same reporting system showed that NF-κB-mediated cellular response to AgNPs is cell-type specific and depends on its basal activity.^42^ Our study shows for the first time that activation of NF-κB-mediated monocyte/macrophage response to NPs, is dependent on their size, metal type and presence or lack of modification.

Most nanoparticles show a tendency to aggregate in biological solutions, thereby leading to formation of large sized aggregates. Thus, the TA-Ag/AuNPs used in this study may form aggregates in biological fluids such as mucus. However, by formation of aggregates with viral antigens, TA-modified nanoparticles may help to internalize viral antigens and facilitate antigen presentation to T and B cells. Several authors have suggested that the size and surface charge of NPs may play a crucial role in the antigen presentation and dendritic cell-mediated antigen uptake, thereby promoting cell-mediated immunity.^7–10^,^17^ We have previously shown in vitro using murine primary DCs and JAWS II cell lines that dendritic cells exposed to non-toxic doses of tannic acid-modified small, medium, and large Ag/AuNPs mixed with HSV-2 antigens decreased major histocompatibility complex (MHC) class I expression and upregulated MHC class II; furthermore.^17^ The expression of MHCII is crucial for antigen presentation by APC to naive T cells, followed by activation of the adaptive immune response.

Considering the results obtained previously and in vitro, we decided to determine how size, metal type, and tannic acid modification influence development of the specific immune response in a mouse model of HSV-1 infection. We found that treatment with all types of NPs significantly decreased the viral titers early during infection in TGs. During the symptomatic phase of infection (day 7), treatment with tannic acid-modified nanoparticles of all types and sizes resulted in lower viral titers and higher infiltration of NK1.1. cells in the TGs and cytotoxic CD8+ T cells in both the brain and the TGs. Tannic acid-modified NPs, both Au and Ag, induced a better antiviral CD8+ T cell response in TGs (AgNPs) and brains (AuNPs), as well as high numbers of activated and virus-specific cytotoxic T cells (Figure 7). It is possible that APCs, such as monocytes and dendritic cells, recognize tannic acid-modified nanoparticle/virus aggregates as foreign antigens, engulf and process them, and then present them to effector immune cells via the MHC in a much better manner than weakly immunogenic viral antigens. This effect was not observed for the unmodified Ag/AuNPs because they did not form aggregates with HSV. Furthermore, treatment with TA-modified NPs resulted in a better profile of antiviral cytokines and chemokines (IFN-gamma, CXCL9, and CXCL10) detected in tissues where HSV-1 establishes latency, such as TGs and brains. This effect was especially visible for 30 nm AgNPs, for which a lack of tannic acid modification actually decreased the expression of antiviral cytokines and chemokines.

Upon skin and mucosal infection, HSV-1 enters epidermal DCs and Langerhans cells, causing non-productive or abortive infection, ending with both cell types becoming apoptotic by 18 h post-infection.^43^ HSV is next transferred from apoptotic LCs to dermal DCs and macrophages in the process termed the “viral relay” and the viral antigens are cross-presented to CD8+ T cells in lymph nodes.^43^,^44^ In our study, application of all tested NPs to the nasal cavities of uninfected mice induced the infiltration of both monocytes and LCs. Interestingly, treatment of early HSV-1 nasal infection with both TA-modified and unmodified 5 and 30 nm AgNPs led to infiltration of inflammatory, activated monocytes within the infected nasal mucosa; however, treatment with tannic-acid-modified Ag/AuNPs led to significant upregulation of IFNs type I, CXCL9, and IL-1β expression within the nasal mucosa early during infection. This indicates that TA plays a significant role in directing the antiviral response early during infection.

Exposure to nanomaterials may lead to inflammation by activating different cell signaling pathways, leading to further release of pro-inflammatory mediators, such as TNF-α, IL-1, β and IL-6. Nanoparticle-induced inflammation may be partially caused by the release of (toxic) metal ions in a process known as NP dissolution.^45^,^46^ The most accepted toxicity mechanism of nanoparticles, especially AgNPs, is the Trojan-horse mechanism, in which particles taken up via endocytosis are subsequently degraded in the lysosome.^45^,^46^ Next, Ag^0^ is oxidized to Ag+, which leads to the production of toxic reactive oxygen species (ROS). Ag+ ions also interfere with the respiratory chain, leading to the formation of additional ROS.^45^,^46^ In our study, increased infiltration of monocytes within the nasal cavity and TGs was observed in tissues subjected to AgNPs of both sizes, TA-modified and unmodified. Interestingly, at 7 d p.i., TGs of mice treated with all types of TA-Ag/AuNPs showed significantly fewer inflammatory monocytes, but more infiltration of Langerhans cells, responsible for HSV antigen uptake and further presentation to immune-competent cells. Therefore, modification with tannic acid may modulate inflammation induced by nanoparticles. Tannic acid was found to inhibit IL-1β secretion by macrophages in an NLRP3-dependent manner^47^ and to modulate EGF-R/Jak2/STAT1/3 and P38/STAT1/p21Waf1/Cip1 pathways.^48^ Anti-inflammatory activity of tannic acid has been confirmed by many studies, although its influence on other signaling pathways is not clear. Furthermore, the measurement of Ag and Au in the exposed tissues clearly indicates that the dissolution of Ag nanoparticles must have occurred. Tannic acid probably caused stronger dissolution or removal from the organism, as we detected very little Ag in mice treated with TA-modified AgNPs of both sizes compared to unmodified AgNPs (Figure 10). Concerning Au, we observed much higher deposition of this element both within the respiratory tract and in the olfactory bulb (OB) and the brain (Figure 10). Patchin et al (2016) demonstrated the ability of unmodified Ag (20 and 110 nm) to be deposited, translocated, and retained from the nose to the olfactory bulbs at levels sufficient to cause microglial activation. The study detected microglia activation and Ag in OB as long as 56 days after inhalation lasting 6h.^49^ Here, we instilled much lower doses of AgNPs or AuNPs, as the single dose was 0.4 μg/mouse (total dose 0.8 μg/mouse). Lee et al determined the extrapulmonary translocation and elimination of AgNPs (10.86 nm) and AuNPs (10.82 nm) in male rats over 28 days (6-h/day) using a nose-only inhalation system. AuNPs were found in many organs (liver, kidney, spleen, olfactory bulb, and brain) and showed a slow rate of elimination.^50^ Ag was translocated to the extrapulmonary tissues and rapidly eliminated from the tissues except the brain and olfactory bulb.^50^ Hence, our results confirm that tannic acid modification may also increase the rate of Ag dissolution and make its use even safer. Additionally, intranasal instillation with AgNPs is much safer than intravenous instillation, as only a small fraction of intratracheal-instilled ultrafine particles can pass rapidly into systemic circulation.^51^ Taking into account the fact that TA-AuNPs were detected in our study within the brain, which could lead to inflammation, as shown by Patchin et al,^48^ our data suggest that AuNPs can be internalized by microglia and help to induce an immune response against HSV-1.

## Conclusion

Here, we demonstrated the role of interactions between the size, material, surface modification, and antiviral activity of NPs against HSV-1 infection in vitro and in vivo. Experiments revealed that tannic acid modification of both AgNPs and AuNPs allows to target and inactivate HSV-1 viral particles, although the mechanism seems to be different depending on the nanoparticle size and metal type. Treatment of the mucosal tissues at the early stage of HSV-1 infection helps to modulate specific and effective antiviral immune response by attracting cytotoxic lymphocytes and inducing the production of antiviral cytokines and chemokines. Furthermore, tannic acid modification is helpful for the removal of nanoparticles from the respiratory tract, which increases the safety of nanoparticle applications to treat infections.
